# Investigation of HNF-1B as a diagnostic biomarker for pancreatic ductal adenocarcinoma

**DOI:** 10.1186/s40364-018-0139-6

**Published:** 2018-07-27

**Authors:** Michelle X. Yang, Ryan F. Coates, Abiy Ambaye, Juli-Anne Gardner, Richard Zubarick, Yuan Gao, Joan Skelly, James G. Liu, Mari Mino-Kenudson

**Affiliations:** 1 Department of Pathology and Laboratory Medicine, University of Vermont Medical Center, 111 Colchester Avenue, Burlington, VT USA; 2 Gastroenterology, University of Vermont Medical Center, 111 Colchester Avenue, Burlington, VT USA; 3 Department of Gastrointestinal Surgery, Nanjing Medical University affiliated Changzhou 2nd People’s Hospital, Changzhou, Jiangsu China; 4 University of Vermont Medical Biostatistics Department, Burlington, VT USA; 5 Applied Pathology Systems, Worcester, MA USA; 6 Department of Pathology, Massachusetts General Hospital, Boston, MA USA; 7 Present address: Department of Pathology, University of Massachusetts Medical Center, 1 Innovation Drive, Worcester, MA 01605 USA

**Keywords:** HNF-1B, Pancreatic, Pancreaticobiliary, Adenocarcinoma, Tissue microarray, Immunohistochemistry

## Abstract

**Background:**

Diagnosing pancreatic ductal adenocarcinoma (PDAC) in the setting of metastasis with an unknown primary remains very challenging due to the lack of specific biomarkers. HNF-1B has been characterized as an important transcription factor for pancreatic development and was reported as a biomarker for clear cell subtype of PDAC.

**Methods:**

To investigate the diagnostic role of HNF-1B for PDAC, we used tissue microarray (TMA) and immunohistochemistry (IHC) to characterize HNF-1B expression in a large cohort of carcinomas, including 127 primary PDACs, 47 biliary adenocarcinomas, 17 metastatic PDACs, and 231 non-pancreaticobiliary carcinomas.

**Results:**

HNF-1B was expressed in 107 of 127 (84.3%) of PDACs, 13 of 15 (86.7%) of cholangiocarcinomas, 13 of 18 (72%) of ampullary carcinomas, and 13 of 14 (92.9%) of gallbladder adenocarcinomas. Notably, HNF-1B was expressed in 16 of 17 (94.1%) of metastatic PDACs. Among the non-pancreaticobiliary cancers, HNF-1B was expressed in ~ 77% clear cell carcinomas of the kidney and ovarian clear cell carcinomas. Gastroesophageal, lung, and prostate adenocarcinomas occasionally expressed HNF-1B in up to 37% cases. HNF-1B was completely negative in hepatocellular, colorectal, breast, and lung squamous cell carcinomas. The sensitivity, specificity, positive predictive value, negative predictive value, and accuracy of HNF-1B for primary pancreaticobiliary carcinoma is 84, 68, 66, 85, and 75%, respectively. HNF-1B expression was not significantly associated with overall survival in patients with PDAC, but tumor size ≥2 cm and high tumor grade were significantly associated with worse overall survival in multivariate analyses.

**Conclusions:**

HNF-1B may be used in surgical pathology to aid the diagnosis of metastatic pancreatic and biliary carcinoma with a panel of other markers to exclude lung, kidney, prostate, and Müllerian origins.

## Background

Pancreatic ductal adenocarcinoma (PDAC) consists of approximately 85% of cancers arising in the pancreas, and is one of the most lethal malignancies in the world. Despite new generations of neoadjuvant and adjuvant therapies, the 5-year overall survival rate remains less than 5%, and patients with PDAC often present with metastatic disease of an unknown primary [1, 2]. An accurate diagnosis of PDAC on biopsy specimens remains challenging due to the lack of specific biomarkers [3, 4]. Investigating additional markers to improve the diagnosis of PDAC is of paramount important in daily practice for surgical pathologists.

Hepatocyte nuclear factor 1B (HNF-1B) has been well-characterized as one of the transcription factors involved in the early development of liver, pancreas, and kidney [5–7]. In animal models, HNF-1B gene was required for the morphogenesis of both ventral and dorsal pancreatic buds [8, 9]. In human subjects, mutations in HNF-1B caused severe pancreatic agenesis or hypoplasia, maturity-onset diabetes of the young (MODY) type 5, multicystic renal dysplasia, and hepatobiliary tract and Müllerian tract abnormalities [10–14]. In human adenocarcinomas, HNF-1B was highly expressed in ovarian clear cell carcinomas and has been recognized as a useful molecular biomarker for this entity [15–17]. Interestingly, a recent study showed that PDAC with clear cell morphology strongly expressed HNF-1B, in contrast to the conventional type PDAC with only weak (61%) to moderate (24%) staining [18]. Due to the essential role of HNF-1B in pancreatic development, we hypothesized that HNF-1B was expressed in all cancers arising from the pancreatic ductal epithelium regardless of the histomorphology, and its expression may serve as a diagnostic marker of these cancers. Using immunohistochemistry (IHC) and tissue microarray (TMA), we investigated HNF-1B protein expression in 127 primary PDACs, 47 biliary tract adenocarcinomas, 17 metastatic PDACs, and 231 common non-pancreaticobiliary carcinomas, and calculated its sensitivity and specificity. The utility of HNF-1B to aid the diagnosis of pancreaticobiliary adenocarcinoma was discussed.

## Methods

### Study population

A total of 127 primary PDAC resections and 17 known metastatic PDACs were retrospectively retrieved from formalin fixed paraffin embedded (FFPE) blocks. Among the 127 primary PDACs, 10 cases received neoadjuvant therapy and 112 cases had negative resection margins (R0). A total of 85 cases had complete survival data with at least 2 years of follow-up after resection. Among the 17 metastatic PDACs, metastatic sites included liver (*N* = 13), celiac lymph nodes (*N* = 2), peritoneum (*N* = 1), and bone (*N* = 1). In addition, 47 adenocarcinomas from the biliary tract and 231 non-pancreaticobiliary carcinomas that morphologically mimic PDAC (mimickers) were also evaluated for comparison, including those of the ampulla, intrahepatic and extrahepatic biliary tract, gallbladder, colorectal, esophagus, stomach, hepatocellular carcinoma, lung (both adenocarcinoma and squamous cell carcinoma), bladder (urothelial carcinoma), breast (ductal and lobular), kidney (mainly clear cell carcinoma), prostate, ovarian surface epithelial, and endometrial. This study was approved by our Institutional Review Board (IRB # 17–0009).

### Histological evaluation and tissue microarray (TMA) construction

All tumor slides of the 127 primary PDAC resections were reviewed, and the size of the tumor, tumor (pT) and nodal (pN) stages, tumor grade, tumor morphology (cytoplasmic clearing), lymphovascular invasion (LVI), perineural invasion (PNI), and resection margin status were extracted from the electronic pathologic record. The death status were extracted from the Tumor Registry data set. The final stage of PDAC was diagnosed in accordance with the American Joint Committee on Cancer (AJCC), 7th edition [19]. Two-millimeter core TMAs were constructed with two cores each from the FFPE tumor tissue or adjacent non-neoplastic pancreas (as control) of primary PDAC resections or 278 non-pancreatic cancers (Beecher Instruments Inc., Sun Prairie, WI). Eleven of 17 metastatic PDACs were also included in the duplicated 2-mm core TMA, and the remaining 6 metastatic PDACs were biopsies that were mounted onto individual slides.

### Immunohistochemistry (IHC)

Polyclonal anti-HNF-1B (Sigma, St. Louis, MO, HPA002083, 1:200 dilutions) was validated in non-neoplastic pancreatic tissue sections. Antigen retrieval was obtained for HNF-1B in H1 buffer (Leica Biosystems, Buffalo Grove, IL) for 10 min, All IHCs were performed in Leica BOND-III automated IHC stainer. Localization of staining – nuclear, cytoplasmic and/or membranous – was recorded in each case, and the case was recorded as positive if any amount of tumor cells had any pattern of HNF-1B expression. A two-tier scale was applied to all positive cases for HNF-1B: “strong”, if the stain was clearly visualized at 20× low magnification, and “weak”, if the stain was clearly visualized at 100× magnification with less intensity.

As comparison, a monoclonal antibody (Abnova, clone CL0374, 1:200 dilution) was validated under the same conditions mentioned above and showed nuclear reactivity in normal pancreatic ductal epithelium, but not in the acinar cells or islet cells, which was consistent with the result of polyclonal antibody. All cancer TMAs were investigated with the HNF-1B expression using the polyclonal antibody.

### Statistical analysis

All demographic and clinicopathological measures were screened for ranges and appropriate codes. Eighty five of the 127 primary PDAC patients had complete demographic and clinicopathological measures and had survival data with at least 2 years of follow-up after the resection. For these 85 patients, descriptive statistics for these measures were calculated. The association between HNF-1B protein expression and clinicopathological features was examined using 2 × 2 contingency tables with Fisher’s extract tests. Univariate Cox proportional hazard function models were used to examine the association of each of the clinicopathological measures with overall survival. The significant measures were included in a stepwise modeling procedure to determine a final model. The expression rate of HNF-1B in PDAC was compared to the rate in each of the other cancer types using Fisher’s exact tests. The sensitivity, specificity and other measures of HNF-1B in classifying pancreaticobiliary and non-pancreaticobiliary carcinomas were calculated with 95% confidence intervals. All statistical analyses were conducted using SAS 9.3 software (SAS Institute, Cary NC). Statistical significant was determined by *P* < .05.

## Results

### Demographics and clinicopathologic features of PDAC

Among the 85 primary PDAC patients with complete clinicopathologic data, the mean age was 65 years (SD = 10), 55 were male (65%) (Table 1). Sixty eight (80%) had positive HNF-1B expression. Fisher’s exact tests found no significant associations between HNF-1B expression and the clinicopathologic parameters. In univariate analysis, only tumor size ≥2 cm (*P =* .03) and high tumor grade (*P* = .02) were significantly associated with worse overall survival. HNF-1B protein expression did not demonstrate significant association with the overall survival. In multivariate analysis, tumor size ≥2 cm (*P* = .03) and high tumor grade (*P* = .02) remained significantly associated with worse overall survival (Table 2).

**Table 1:** Demographics and clinicopathological parameters in patients with PDAC

Variables	PDAC (*N* = 85)
Age: Mean ± SD (years)	65 ± 10
Gender	
Male	55 (65%)
Female	30 (35%)
Tumor size	
≤ 2 cm	18 (21%)
> 2 cm	67 (79%)
Tumor location	
Head	63 (74%)
Tail	22 (26%)
LN metastasis	54 (64%)
LVI	40 (48%)
PNI	61 (72%)
No neoadjuvant	75 (88%)
Stage	
Stage I, II	24 (28%)
Stage III, IV	61 (72%)
Tumor grade	
Low grade (1/2)	56 (66%)
High grade (3)	29 (34%)

Abbreviations: *PDAC* pancreatic ductal adenocarcinoma;

*SD* standard deviation, *LVI* lymphovascular invasion;

*LN* lymph node, *PNI* perineural invasion

**Table 2:** Overall survival and clinicopathological variables in 85 patients with PDAC

Variables	Hazard ratio (95% CI)	*p*-value
Univariate analysis		
Age (< 65 vs ≥65)	0.98 (0.59–1.62)	.94
Gender (male vs. female)	1.13 (0.68–1.88)	.65
Tumor size (< 2 cm vs. ≥ 2 cm)	2.11 (1.07–4.16)	**.03**
Stage (I/II vs. III/IV)	0.74 (0.44–1.26)	.28
Grade (low vs. high)	1.84 (1.11–3.05)	**.02**
PNI	1.18 (0.70–2.01)	.54
LVI	1.46 (0.90–2.40)	.13
LN metastasis	1.31 (0.77–2.21)	.32
HNF-1B (negative vs. positive)	1.34 (0.70–2.57)	.38
No neoadjuvant	1.14 (0.41–1.14)	.80
Multivariate analysis		
Tumor size (< 2 cm vs. ≥ 2 cm)	2.10 (1.06–4.16)	**.03**
Grade (low vs. high)	1.83 (1.10–3.06)	**.02**

Abbreviations: PDAC, pancreatic ductal adenocarcinoma; VS, versus;

*SD* standard deviation, *LVI* lymphovascular invasion;

LN lymph node, *PNI* perineural invasion

Bold: Statistically significant

### HNF-1B expression in non-neoplastic pancreatic and biliary epithelium

**Fig. 1:**
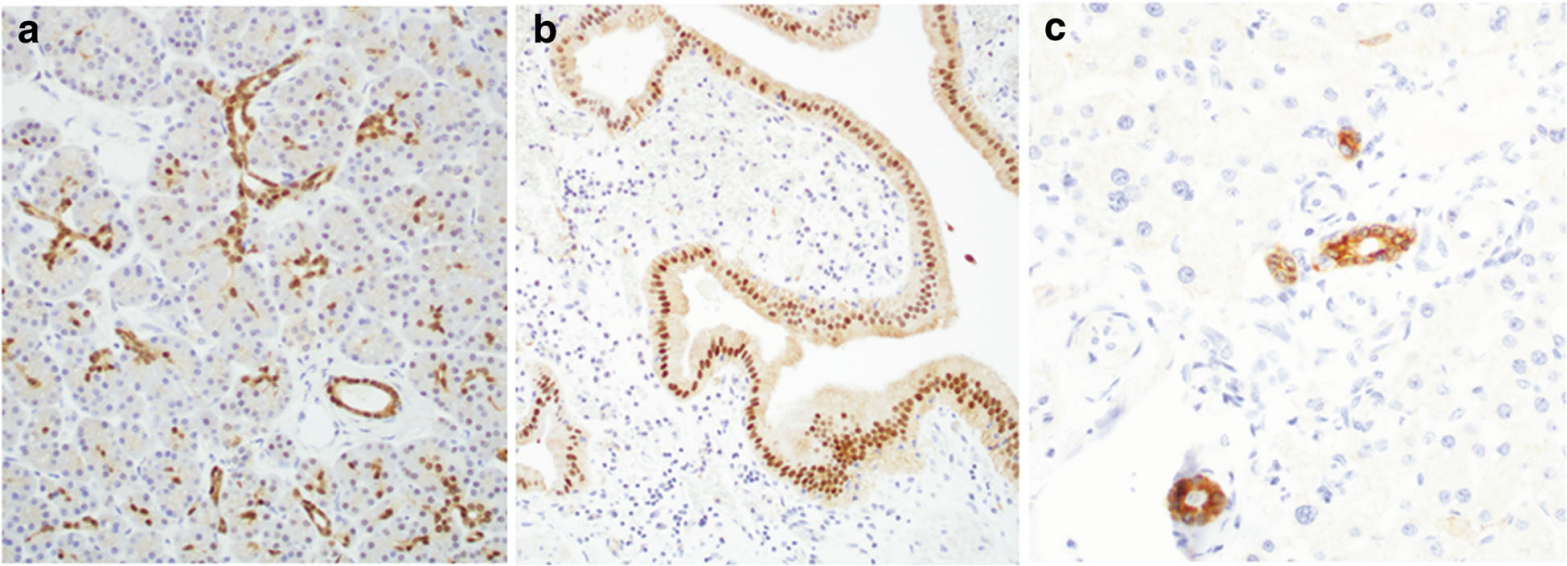
HNF-1B expression pattern by IHC in non-neoplastic pancreaticobiliary epithelium, including (**a**) Pancreas, (**b**) Gallbladder, and (**c**) intrahepatic bile duct. Original magnification, 200×

In non-neoplastic adult pancreas, HNF-1B was expressed in the ductal epithelium and centroacinar ductal cells with predominant nuclear and faint cytoplasmic staining (Fig. 1a). Since HNF-1B is also a transcription factor for liver development, we investigated HNF-1B immunostaining in normal liver and gallbladder. Interestingly, HNF-1B showed predominant nuclear staining in the gallbladder epithelium (Fig. 1b), in contrast to a predominant cytoplasmic staining in non-neoplastic intrahepatic ductal and extrahepatic ductal epithelium (Fig. 1c).

### Morphological variation of primary and metastatic PDAC

**Fig. 2:**
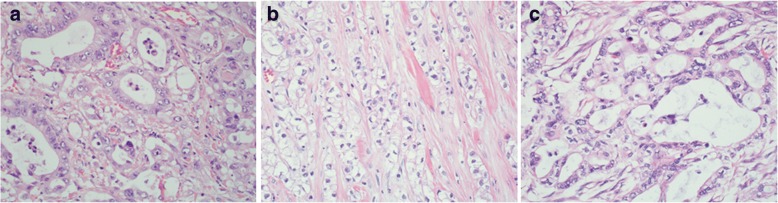
Histomorphological variation of PDAC on hematoxylin and eosin (HE) staining, including (**a**) conventional, (**b**) clear cell variant, and (**c**) mixed features. Original magnification, 400×

In a previous study, Kim et al. reported that HNF-1B was strongly expressed in PDAC with cytoplasmic clearing and only weakly to moderately expressed in PDAC with conventional histomorphology.^18^ We reviewed the morphology specifically the cytoplasmic clearing for all the PDAC cases in this cohort and separated them into 3 categories: 1) Conventional- if > 95% of tumors cells demonstrated no cytoplasmic clearing (Fig. 2a); 2) Prominent clearing- if > 75% tumor cells demonstrated cytoplasmic clearing (Fig. 2b); and 3) mixed features- if 5–75% tumor cells demonstrated cytoplasmic clearing (Fig. 2c). Among the 127 primary PDACs, 84 cases (66.1%) were conventional PDAC, and 43 cases (33.9%) showed variable cytoplasmic clearing in the tumor cells, including 10 cases (7.9%) with prominent clearing, and 33 cases (26%) with mixed features. In 17 metastatic PDACs, 1 case showed prominent cytoplasmic clearing, 2 cases had mixed features, and the remaining showed conventional morphology.

### Variable expression pattern of HNF-1B in primary and metastatic PDAC

**Fig. 3:**
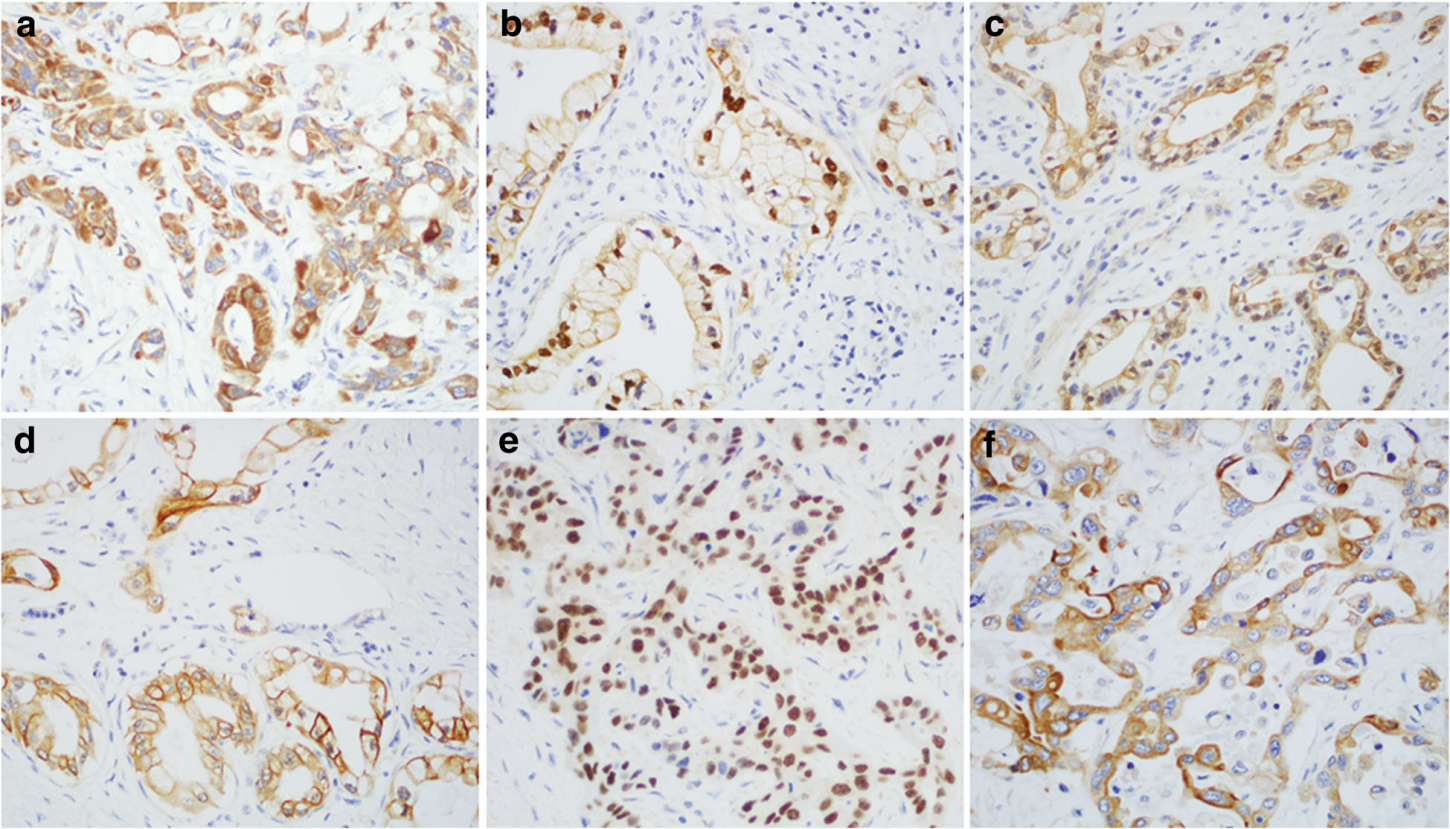
Variable expression pattern of HNF-1B by IHC in primary and metastatic PDACs. (**a**) Primary PDAC with cytoplasmic staining, (**b**) Primary PDAC with nuclear staining, (**c**) Primary PDAC with nuclear and cytoplasmic staining, (**d**) Primary PDAC with cytoplasmic and membranous staining, (**e**) Metastatic PDAC with nuclear staining, and (**f**) Metastatic PDAC with cytoplasmic staining. Original magnification, 400×

Although HNF-1B was characterized as a nuclear transcription factor, HNF-1B was expressed in a total of 107 (84.3%) PDACs with multiple staining patterns, including cytoplasmic staining in 64 (59.8%) cases (Fig. 3a), nuclear staining in 32 (29.9%) cases (Fig. 3b), nuclear and cytoplasmic staining in 6 (5.6%) cases (Fig. 3c), and cytoplasmic and membranous staining in 5 (4.7%) cases (Fig. 3d). Among the 107 HNF-1B positive PDAC cases, 90 cases (70.9%) showed strong staining, while 17 (13.4%) showed weak staining pattern. There was no significant association of HNF-1B staining pattern or intensity with cytoplasmic clearing of the tumor cells.

Sixteen of 17 (94.1%) metastatic PDACs were positive for HNF-1B, including 13 cases (76.5%) with strong staining and 3 cases (17.6%) showed weak staining. Interestingly, 12 metastatic PDACs (70.6%) showed predominantly nuclear immunoreactivity with or without cytoplasmic staining (Fig. 3e, f).

### HNF-1B expression in non-pancreatic carcinomas

In order to investigate whether HNF-1B protein expression was restricted to the adenocarcinomas of pancreatic ductal origin, HNF-1B IHC was performed on a total of 278 common carcinomas from other organ systems, including cholangiocarcinoma (intrahepatic and extrahepatic biliary tracts), ampullary region, gallbladder, colorectum, distal esophagus, stomach, hepatocellular, lung adenocarcinoma, lung squamous cell carcinoma, breast, prostate, ovary, uterus, bladder urothelial, and kidney.

Not surprisingly, HNF-1B was strongly immunoreactive with nuclear and/or cytoplasmic patterns in 13 of 15 (86.7%) intrahepatic and extrahepatic cholangiocarcinomas, 13 of 18 (72.2%) ampullary adenocarcinomas, and 13 of 14 (92.9%) gallbladder adenocarcinomas. In addition, strong nuclear immunoreactivity of HNF-1B was observed in 16 of 21 (76.1%) clear cell carcinomas of the kidney, 10 of 14 (71.4%) ovarian clear cell carcinomas, 6 of 24 (25%) lung adenocarcinomas, and 4 of 15 (26.7%) prostate adenocarcinomas. Weak nuclear with or without cytoplasmic HNF-1B expression was seen in 3 of 11 (27.3%) esophageal adenocarcinomas, 9 of 14 (64.3%) stomach adenocarcinomas, 7 of 18 bladder urothelial (38.9%), and 10 of 21 (42.8%) non-clear cell type Müllerian carcinomas. HNF-1B was completely negative in all colorectal cancer, breast cancer, hepatocellular carcinoma, and lung squamous cell carcinoma (Table 3). Fisher’s exact tests showed significantly lower HNF-1B expression rate in the colorectal, hepatocellular, esophageal, lung, breast, bladder, prostate, and uterine carcinomas (*P* < .001, Table 3).

**Table 3:** Immunoreactivity of HNF-1B in PDAC and non-pancreatic carcinomas

Cancer type	Total No.	HNF-1B +	Stain pattern	Fisher’s exact *p*-value
Pancreatic primary	127	107 (84.3%)	C, N, M	ref
Cholangiocarcinoma	15	13 (86.7%)	C, N, M	.81
Ampullary	18	13 (72%)	C, N, M	.21
Gallbladder	14	13 (92.9%)	C, N, M	.69
Colorectal	39	0	–	<.001
Hepatocellular	20	0	–	<.001
Esophagus	11	3 (27.3%)	C, N	<.001
Stomach	14	9 (64.3%)	C, N	.13
Lung adenocarcinoma	24	6 (25%)	C, N	<.001
Lung Squamous cell	10	0	–	<.001
Breast	20	0	–	<.001
Bladder	18	7 (38.9%)	C, N	<.001
Prostate	15	4 (26.7%)	C, N	<.001
Kidney	21	16 (76.2%)	N	.35
Ovary	18	14 (77.8%)	N, C	.50
Uterus	21	10 (47.6%)	N, C	<.001
Metastatic PDAC	17	16 (94.1%)	N, C	
Total No.	422			

Abbreviations: PDAC, pancreatic ductal adenocarcinoma;

*SCC* squamous cell carcinoma, *C* cytoplasmic, *N* nuclear, *M* membranous

### Sensitivity and specificity of HNF-1B in pancreaticobiliary carcinomas

Since we observed that HNF-1B was expressed in the majority of carcinomas arising from the pancreatic and biliary epithelium, we calculated the sensitivity, specificity, positive predictive value, negative predictive value, and accuracy with 95% confidence interval (95% CI) of HNF-1B for all primary pancreaticobiliary carcinomas in comparison to non-pancreaticobiliary carcinomas. The results were summarized in Table 4. Overall, HNF-1B showed high sensitivity (84%) and high negative predictive value (85%) for primary pancreaticobiliary carcinomas with moderate specificity and accuracy (68 and 75%, respectively).

**Table 4:** Sensitivity and specificity of HNF-1B in pancreaticobiliary carcinomas

Measure	Proportion	95% CI
Sensitivity	0.84	(0.79, 0.90)
Specificity	0.68	(0.62, 0.74)
Positive Predictive Value	0.66	(0.60, 0.73)
Negative Predictive Value	0.85	(0.80, 0.90)
Accuracy	0.75	(0.71, 0.79)

Abbreviation: *CI* confidence interval

## Discussion

We investigated the protein expression pattern of HNF-1B in 127 primary PDACs, 47 biliary adenocarcinomas, 17 metastatic PDACs, and 231 other common carcinomas that may mimic PDAC. HNF-1B was highly expressed in adenocarcinomas along the pancreaticobiliary tract, including PDAC, intrahepatic and extrahepatic cholangiocarcinomas, ampullary adenocarcinomas, and gallbladder adenocarcinomas with nuclear and/or cytoplasmic staining pattern. Importantly, HNF-1B expression was expressed in 94.1% of metastatic PDAC with predominantly nuclear staining.

KRAS mutation is a frequent molecular abnormality that is identified in up to 90% of PDACs [20]. Interestingly, a recent study showed that mutated KRAS can induce abnormal regulations of pancreatic transcription factors including HNF-1B, which in turn causes abnormal cell growth and proliferation that leads to pancreatic cancer [21]. These findings were consistent with the fundamental pathophysiological role of HNF-1B in the pancreaticobiliary system. The other group has also reported cytoplasmic and/or nuclear expression of HNF-1B in PDAC [18]. Although transcription factors are translated in the cytoplasm, they are generally translocated into the nucleus to regulate downstream target genes in active physiological state. During inactive regulation or with aberrantly excessive expression, transcription factors may form complex with other proteins and remain in the cytoplasm and/or cell membrane. This might explain why HNF-1B showed variable nuclear, cytoplasmic and/or membranous staining patterns in PDAC and other carcinomas.

Among non-pancreaticobiliary carcinomas, clear cell carcinomas of the kidney showed predominantly nuclear HNF-1B expression, while Müllerian origin, including ovarian and endometrial clear cell carcinoma showed nuclear and/or cytoplasmic HNF-1B expression. The expression of HNF-1B in carcinomas of kidney and Müllerian origin also indicates its pathophysiological role in these organs. Interestingly, silencing of HNF-1B expression secondary to promoter methylation appears to promote disease progression via epithelial-to-mesenchymal transition in both prostate and ovarian cancers [22]. In contrast to the association of HNF-1B with poor prognosis in PDAC observed by Kim et al. [18], HNF-1B seemed to have tumor-suppressor role in both prostate and kidney cancers [22, 23]. Thus, a biological role(s) of HNF-1B as well as the significance of its aberrant cytoplasmic and membranous expressions in different types of cancer needs to be investigated in the future.

## Conclusions

Our data suggested that HNF-1B may serve as a useful diagnostic biomarker for tumors of the pancreaticobiliary origin with high sensitivity and negative predictive value, but moderate specificity and accuracy for these tumors. Since HNF-1B can also be expressed less frequently in variable tumors of the non-pancreaticobiliary origin, especially of the kidney, Müllerian tract, lung, gastroesophageal, bladder, and prostate carcinomas, the concurrent use of other markers such as TTF-1, PAX-8, WT-1, CAIX, NKX3.1, p40, and PSA as a panel to rule out other organ primaries, and correlation with imaging studies and/or endoscopic findings are important to refine the diagnosis.

## Abbreviations

CAIX: Carbonic anhydrase 9
FFPE: Formalin fixed paraffin embedded
HE: Hematoxylin and eosin
HNF-1B: Hepatocyte nuclear factor 1B
IHC: Immunohistochemistry
MODY: Maturity-onset diabetes of the young
NKX3.1: NK3 Homeobox 1
PAX-8: Paired box gene 8
PDAC: Pancreatic ductal adenocarcinoma
PSA: Prostate specific antigen
SD: Standard deviation
TMA: Tissue microarray
TTF-1: Thyroid transcription factor 1
WT-1: Wilms’ tumor 1
